# Systematic Analysis of Palatal Rugae Pattern for Use in Human Identification between Two Different Populations

**Published:** 2017-05

**Authors:** Radhika Kalyani KOMMALAPATI, Deepthi KATURI, Kiran Kumar KATTAPPAGARI, Lalith Prakash Chandra KANTHETI, Rajasekhar Babu MURAKONDA, Chandra Sekhar POOSARLA, Ravi Teja CHITTURI, Sridhar Reddy GONTU, Venkata Ramana Reddy BADDAM

**Affiliations:** 1. Dept. of Oral Pathology and Microbiology, Sibar, Institute of Dental Sciences, Guntur, Andhra Pradesh, India; 2. Dept. of Oral Pathology and Microbiology, St. Joseph’s Dental College, Elur, Andhra Pradesh, India; 3. Practitioner, Guntur, Andhra Pradesh, India; 4. Sibar Health and Research Foundation, Sibar Institute of Dental Sciences, Guntur, Andhra Pradesh, India

**Keywords:** Forensic dentistry, Palatal rugae, Rugoscopy, Human identification

## Abstract

**Background:**

Forensic odontology plays important role in identification of human remains in mass disasters such as air crash, tsunami, and accidents. Palatal rugae act as an ideal requisite for human identification as they are present in all victims and are resistant to changes such as aging and trauma. The study aimed to analyze differences in shape and number of palatal rugae in population from Andhra Pradesh and Telangana states.

**Methods:**

This study was conducted in SIBAR institute of Dental Sciences, Guntur, India in 2012 on 200 subjects, gender matched and equally divided as 100 from Andhra Pradesh and 100 from Telangana states. Rugae were studied using Kapali.S classification. Association between rugae shape and gender variation between the two populations were tested by chi-square analysis and student *t-*test.

**Results:**

Average number of rugae was more (11.84±3.03) in subjects of Andhra Pradesh, compared to Telangana (9.50 ±1.65) population. Males of Andhra Pradesh showed significantly higher mean number of total rugae than in Telangana males. Distribution of total number of different rugae shapes in males and females of both the populations showed significant variation in wavy and circular rugae patterns. Wavy, curved and straight rugae were significantly higher in males and females of Andhra Pradesh compared to Telangana population. Telangana population showed significant increase in circular rugae.

**Conclusion:**

Our study revealed statistically significant variation in shape and total number of rugae between observed populations of Andhra Pradesh and Telangana states.

## Introduction

Forensic Science plays a significant role in ascertaining the circumstances of death and thus acts as the last council of defense. Identification of humans is a prime requisite for certification of death and for personal, social and legal reasons (1). Fingerprints and other visual identification methods have limited because, tissue level changes are associated with time, temperature and humidity. Besides, time and temperature may influence the soft tissue changes (2). Forensic odontology is one of the important aspect for human identification in mass disasters such as tsunami and air crashes. Methods like cheiloscopy, bite mark analysis, rugoscopy, radiographs, photographic study and molecular techniques (Polymerase Chain Reaction for pulp DNA analysis) have been established for human identification in Forensic Odontology (3). Among these methods, palatal rugoscopy/palatoscopy (study of palatal rugae) may be very much useful for identification of human remains. This method is advantageous because of the anatomical location of palatal rugae, protected from external factors by the tongue and buccal mucosa (4, 5).

Palatal rugae are described as anatomical folds or wrinkles, present posterior to the incisive papillae and are called as “Pilca Palatine” or “Rugae Palatine” (6). They are present as ridges on the anterior part of the palatal mucosa on each side of the median palatal raphae and behind the incisive papilla. Embryologically, the rugae core begins to differentiate at the 20^th^ week of intra uterine life (IUL) (7). Histologically, rugae are similar to the adjacent tissue of the palate showing parakeratinized stratified squamous epithelium on a connective tissue base (8). The anatomical location of palatal rugae makes them more resistant to changes. Hence, they may aid as landmarks during orthodontic treatments, cleft palate surgeries, palatal prosthesis fabrication and medico legal identification. Palatal rugoscopy is well established technique in forensic odontology. This could be because of technique simplicity, uniqueness of rugae to an individual, stability of their shape, position, and ability to withstand high temperatures, providing better evidence than others in mass disasters (9). Importance of palatal rugae was established by different authors in different regions and different ethnic populations. The present study aimed to analyze the differences in shape and number of palatal rugae in two different populations of Andhra Pradesh and Telangana states in South India.

The present study aimed to evaluate the predominant rugae pattern in each region as well as to compare gender variation within and in between the two regions.

## Materials and Methods

This cross-sectional study was conducted in the Department of Oral and Maxillofacial Pathology after obtaining approval from Institutional Ethical Committee Board, Guntur, Andhra Pradesh, India

The study subjects were selected from two states of South India, with different geographic location in 2012. The study population consisted of 200 healthy subjects divided as 100 from Andhra Pradesh state and 100 from Telangana. In each group, they were again divided equally into fifty males and fifty females.

### Inclusion Criteria

- People born and brought up in Andhra Pradesh and Telangana regions
- People aged above 18 yr.

### Exclusion Criteria

- Subjects below the age of 18yr,
- Congenital anomalies (cleft palate, cleft lip)/malformations,
- Previous history of orthognathic surgery/orthodontic treatment,
- Subjects who are allergic to impression material,
- Presence of bony and soft tissue protruberences, active lesions, deformity, scars and trauma

**Fig. 1:**
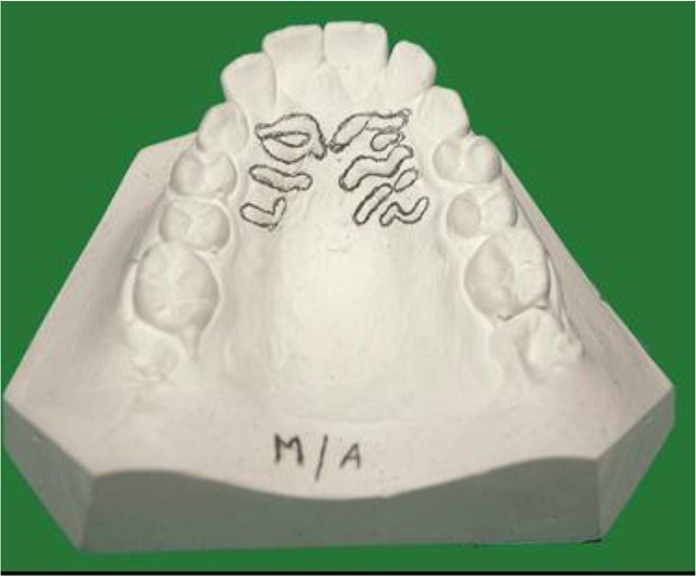
Palatal rugae shapes in Coastal Andhra population

**Fig. 2:**
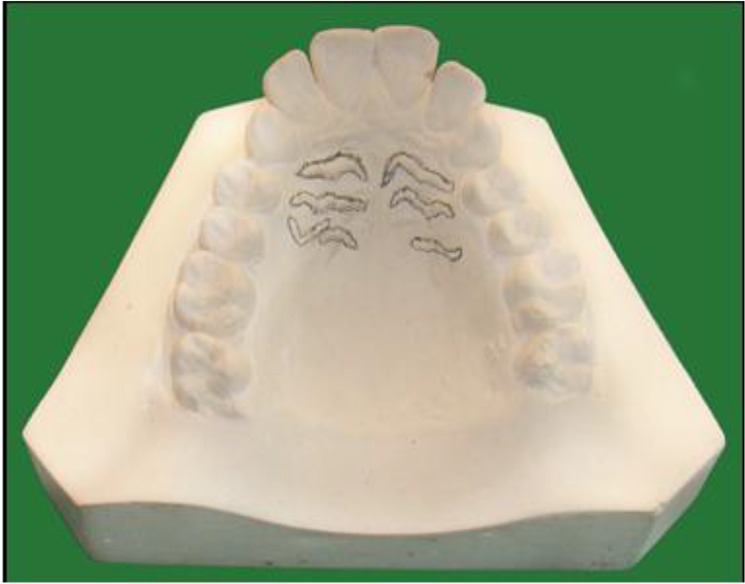
Palatal rugae shapes in Telangana population

After informed consent, maxillary arch impressions of the study subjects were taken using irreversible hydrocolloid materials on perforated metal trays. The metal trays were selected according to arch size and shape. All the manufacturer protocols were followed when mixing water powder ratio. Casts were poured using dental stone mixed on vacuum vibrator to avoid air bubbles. Palatal rugae were highlighted using graphite pencil and magnifying glass under adequate light (Fig. 1, 2).

According to Kapali classification, rugae were divided into (10):

- **Straight** - rugae run directly from their origin to insertion
- **Circular** - rugae which have definite continuous ring
- **Curved** - rugae, which have simple crescent, shape which curve gently.
- **Wavy** - rugae which are serpentine in nature
- **Unification** (Convergent/Divergent) - two rugae are joined at the origin

In this study, all forms of unified and branched rugae were categorized under unification. All rugae were considered for the study irrespective of their length. Inter observer variations were checked out. Association between rugae shape and gender was tested using Chi-square analysis and unpaired t-test using the Statistical Package for Social Sciences (SPSS) ver. 20.0 (Chicago, IL, USA).

## Results

Total number of participants in our study were 200, among these 100 were of Coastal Andhra and 100 were from Telangana with the mean age of 24 yr. gender matched and equally distributed. Genderwise comparison for the mean number of rugae and different rugae shapes was not statistically significant in Andhra population (Table 1).

**Table 1:** Comparison of males and females with respect to mean number of rugae by unpaired t-test (Coastal Andhra samples) and Distribution of total number of different rugae shapes in males and females (Coastal Andhra samples)

	**Mean**	**S.D.**	**Mean**	**S.D.**	
Total	11.48	3.09	12.20	2.96	0.236
Curved	3.54	2.23	3.70	2.02	0.708
Wavy	4.36	1.88	4.96	2.10	0.135
Straight	1.80	1.64	2.00	1.41	0.515
Circular	0.76	1.39	0.88	1.30	0.657
Branched	0.98	1.13	0.66	0.77	0.102
**Rugae shape**	**Male**	**%**	**Female**	**%**	***P*-value**
Curved	47	94.00	48	96.00	0.646
Wavy	49	98.00	50	100.00	0.314
Straight	38	76.00	43	86.00	0.202
Circular	18	36.00	23	46.00	0.309
Branched	28	56.00	25	50.00	0.547

A genderwise comparison of mean number of rugae was not statistically significant in Telangana population also. However, a similar comparison of rugae shapes in Telangana population showed wavy rugae pattern to be more in females and it was statistically significant (*P*: 0.014) (Table 2). The mean number of rugae in males of Andhra population was significantly more compared to that of Telangana (*P*: 0.001). Similarly, the mean value of wavy curved and straight rugae patterns were significantly higher in males of Andhra population (*P*: 0.001).

**Table 2:** Comparison of males and females with respect to mean number of rugae by unpaired t-test (Telangana region samples) and Distribution of total number of different rugae shapes in males and females (Telangana samples)

Variable	**Male (n=50)**	**Female (n=50)**	***P* Value**	**Variable**	**Male (n=50)**	
	**Mean**	**Std.Dev**	**Mean**		**Mean**	
Total	9.42	2.00	9.58	Total	9.42	
Curved	2.32	1.54	2.28	Curved	2.32	
Wavy	2.80	1.91	3.24	Wavy	2.80	
Straight	0.34	0.77	0.26	Straight	0.34	
Circular	3.22	1.58	3.16	Circular	3.22	
Branched	0.72	0.95	0.66	Branched	0.72	
Rugae shape	**Male**	**%**	**Female**	**Rugae shape**	**Male**	***P*-value**
Curved	47	94.00	46	Curved	47	0.695
Wavy	42	84.00	**49**	Wavy	42	0.014
Straight	10	20.00	9	Straight	10	0.798
Circular	46	92.00	46	Circular	46	1.000
Branched	22	44.00	24	Branched	22	0.688

Nevertheless, the mean number of circular rugae in Telangana males was significantly higher than in those of Andhra Pradesh (*P*: 0.001) (Table 3).

**Table 3:** Comparison of Telangana and Coastal Andhra regions with respect to mean number of rugae by unpaired *t*- test (Total samples, n=200)

	**Mean**	**S.D.**	**Mean**	**S.D.**	
Total	9.50	1.65	11.84	3.03	0.00001*
Curved	2.30	1.49	3.62	2.12	0.00001*
Wavy	3.02	1.75	4.66	2.01	0.00001*
Straight	0.30	0.69	1.90	1.53	0.00001*
Circular	3.19	1.46	0.82	1.34	0.00001*
Branched	0.69	0.87	0.82	0.98	0.322

The mean number of rugae and the mean value of straight rugae in females of Andhra Pradesh were significantly more compared to Telangana females (*P*≤0.05). However, the mean number of circular rugae was found to be significantly more in Telangana females (*P*≤0.05).

The comparison of different rugae shapes between the whole selected group of Andhra Pradesh and Telangana populations showed a statistically significant more number of wavy and straight patterns in Andhra population and circular pattern in Telangana population (*P*≤0.05).

## Discussion

Total number of rugae was more in Coastal Andhra when comparing with Telangana. Comparison of mean number of rugae pattern with gender variation in Coastal Andhra and Telangana population did not show any significant, but when compared the different rugae shapes in both male and females in Telangana population wavy rugae pattern was more in Telangana females than males. Telangana population showed significant increase in number of circular patterns compared with Coastal Andhra females than Telangana females.

Dental evidence can be used as a sole method of identifying a dead person (11). Rugoscopy is still in its infancy in the field of forensic odontology. Although there is problem of describing palatal rugae pattern quantitatively and qualitatively due to absence of a single standardized classification, the use of palatal rugae in forensic identification is preferred because of its cost efficiency, relaiability and simplicity. Uniqueness of palatal rugae in individuals has been recognized as an important factor, which helps in providing a potentially reliable source of identification. Several studies done in the past have revealed and have statistically proved that the rugae pattern is highly individualistic and there are racial and gender variations (12).

Different authors for palatal rugae shapes gave different classifications, however, in our study; Kapali classification was used. We did not consider rugae length (primary and secondary rugae) in order to reduce the complexity and to avoid confusion in their categorization (10).

Total number of rugae between males and females of same area did not show any statistical significance (13, 14). In our study, also there was no statistically significant difference in the total number of rugae between males and females of the two populations analyzed individually.

In our study, comparison of different rugae shapes was not statistically significant between males and females of Andhra Pradesh (2). Whereas in Telangana population, wavy rugae shape were found to be significantly more in females (5).

The mean number of primary rugae in Australian Aborigines was higher than that in Caucasians (10). In the present study also, the mean total number of rugae was found to be more in Andhra Pradesh, compared to Telangana population and the findings were statistically significant. Wavy and curved rugae were most prevalent shapes in Southern and Western Indians, which contributed to discriminant function and enabled population identification with 70% accuracy (2). Our study showed that wavy, curved and straight rugae were significantly more in males and females of Andhra Pradesh than in Telangana population.

Our study also showed the total mean number of rugae to be more in males of Andhra Pradesh compared to Telangana, with high statistical significance. Comparison of different rugae shapes between females of Andhra Pradesh and Telangana revealed significantly higher number of curved, wavy and straight patterns in Andhra Pradesh. However, circular rugae were observed to be significantly more in Telangana females than in Andhra Pradesh. A similar study was revealed statistically significant difference in wavy rugae shape in females than in males (15).

Wavy pattern was the predominant one followed by curved, straight, branched and circular rugae (16). These findings are consistent with our present study where wavy rugae were highest in number (26.76%), followed by curved (26.48%), circular (18.73%), straight (14.8%) and branched (13.94%) rugae.

## Conclusion

There were few differences in palatal rugae pattern in both Coastal Andhra and Telangana population. The mean number of rugae was more in Coastal Andhra than Telangana population. When observed wavy pattern was more in Coastal Andhra when compared with Telangana. These observations cannot draw a conclusion for identification tool for ethnicity in different population. It requires further studies with larger sample size to establish these results.

## Ethical considerations

Ethical issues (Including plagiarism, informed consent, misconduct, data fabrication and/or falsification, double publication and/or submission, redundancy, etc.) have been completely observed by the authors.
